# ROS-scavenging materials for skin wound healing: advancements and applications

**DOI:** 10.3389/fbioe.2023.1304835

**Published:** 2023-12-12

**Authors:** Yongkang Dong, Zheng Wang

**Affiliations:** ^1^ Department of Vascular Surgery, The Second Hospital of Jilin University, Changchun, Jilin, China; ^2^ Department of Spine Surgery, The Second Hospital of Jilin University, Changchun, Jilin, China

**Keywords:** reactive oxygen species, oxidative stress, wound healing, biomaterials, skin wound

## Abstract

The intricate healing process of skin wounds includes a variety of cellular and molecular events. Wound healing heavily relies on reactive oxygen species (ROS), which are essential for controlling various processes, including inflammation, cell growth, angiogenesis, granulation, and the formation of extracellular matrix. Nevertheless, an overabundance of reactive oxygen species (ROS) caused by extended oxidative pressure may result in the postponement or failure of wound healing. It is crucial to comprehend the function of reactive oxygen species (ROS) and create biomaterials that efficiently eliminate ROS to enhance the healing process of skin wounds. In this study, a thorough examination is presented on the role of reactive oxygen species (ROS) in the process of wound healing, along with an exploration of the existing knowledge regarding biomaterials employed for ROS elimination. In addition, the article covers different techniques and substances used in the management of skin wound. The future prospects and clinical applications of enhanced biomaterials are also emphasized, highlighting the potential of biomaterials that scavenge active oxygen to promote skin repair. This article seeks to enhance the understanding of the complex processes of ROS in the healing of wounds and the application of ROS-scavenging materials. Its objective is to create novel strategies for effective treatment skin wounds.

## 1 Introduction

Skin wound, caused by physical or heat-related causes and other possible factors, can cause the skin defect and hinder regular bodily functions (Shi et al., 2019). The healing of skin wound is a meticulous and intricate process that involves immune cells, non-immune cells, cytokines, growth factors, and extracellular component (Heher et al., 2018; Liang et al., 2023). Any disturbance in these factors can potentially result in chronic wound healing. The challenging problem of wound healing comes mainly from chronic wounds, especially those skin wounds caused by underlying physiological factors such as diabetes. The process of wound healing involves ongoing formation of new blood vessels, multiplication of cells, and restructuring of the wound tissue. In recent years, growing evidence indicates that reactive oxygen species (ROS) play a significant role in the physiological and pathological aspects of wound healing. ROS is involved in several skin tissue regeneration processes such as the regulation of inflammation, cell proliferation, angiogenesis, granulation, and the formation of extracellular matrix (Xu et al., 2020).

ROS consist of extremely active and oxidizing compounds, which encompass the superoxide anion (O_2–_), hydrogen peroxide (H_2_O_2_), and hydroxyl radical (−OH). Under normal physiological conditions, moderate levels of ROS participate in cellular signaling and immune responses. Nevertheless, under the influence of external factors such as injury, inflammation, or exposure to radiation, the generation of ROS escalates considerably, resulting in oxidative stress within cells and subsequent harm to skin tissues (Khorsandi et al., 2022). The overproduction and buildup of ROS beyond the cellular ability to counteract them obstructs the wound tissue shift from the inflammatory stage to the proliferative stage (Deng et al., 2019; Malone-Povolny et al., 2019). Consequently, wound area is chronically inflamed, resulting in delayed wound healing. Therefore, maintaining the homeostasis of REDOX in cells can prevent abnormal cell growth and immune dysregulation. Multiple research studies have shown that antioxidants have the potential to expedite the process of wound healing, especially when dealing with chronic wounds (Fitzmaurice et al., 2011; Yang et al., 2024). Therefore, the introduction of antioxidant component has emerged as an effective approach to expedite the healing of chronic wounds.

Due to the comprehensive understanding of the relationship between ROS and skin injuries, the use of ROS removal materials to repair skin wounds has attracted great attention. ROS-scavenging materials have the capability to eliminate surplus ROS, relieving cellular and tissue harm caused by oxidative stress and enhancing the recovery of skin wounds. These substances have the ability to trap, counteract, or hinder the production of ROS, thereby restoring internal redox equilibrium, diminishing inflammatory reactions, and promoting cellular growth and rejuvenation. In this review, recent progress in using ROS-scavenging materials for repair skin wounds is presented. The discussion primarily focuses on the correlation between skin wound healing and ROS. Subsequently, the characteristics and applications of different ROS-scavenging materials in skin wound repair are highlighted. In conclusion, this paper discusses the obstacles encountered and the prospect of future applications in the use of ROS-scavenging materials, with the goal of offering useful insights and recommendations for future studies of ROS-scavenging materials.

## 2 Factors that influence wound healing

The skin is the body’s largest organ, comprising the epidermis, dermis, and subcutaneous tissue. The outer layer of the skin acts as a shield, safeguarding against dehydration and external irritations. The dermis provides flexibility, support, and houses blood vessels, nerve fibres, and lymphatic systems. The subcutaneous layer contains adipose tissue and rich vascularization, contributing to temperature regulation (Xu et al., 2020). The process of wound healing is a dynamic and organized process that can be classified into acute and chronic wounds according to their characteristics (Table 1).

**TABLE 1:** Comparison of acute and chronic wounds.

Type of skin wound	Etiology	Wound characteristics	Prognosis	Reference
Acute wound	Caused by mechanical, thermal or chemical damage	There are typical symptoms of pain, inflammation, bleeding and tissue defects	It usually heals within 8–12 weeks through the normal wound healing process	Franz et al. (2000), Nicks et al. (2010), Hurlow and Bowler (2022)
Chronic wound	On the basis of acute wounds, it is accompanied by continuous stimulation of diseases such as diabetes, blood vessel and pressure sores, and bacterial infections	On the basis of acute wounds, it is associated with immune and metabolic disorders, and further impairs vascular structural integrity and tissue regeneration	It gradually heals (12 weeks or more) after the injury and is accompanied by inflammation	Han and Ceilley (2017), Wallace et al. (2019), Bay et al. (2021), Vecin and Kirsner (2023)

**FIGURE 1:**
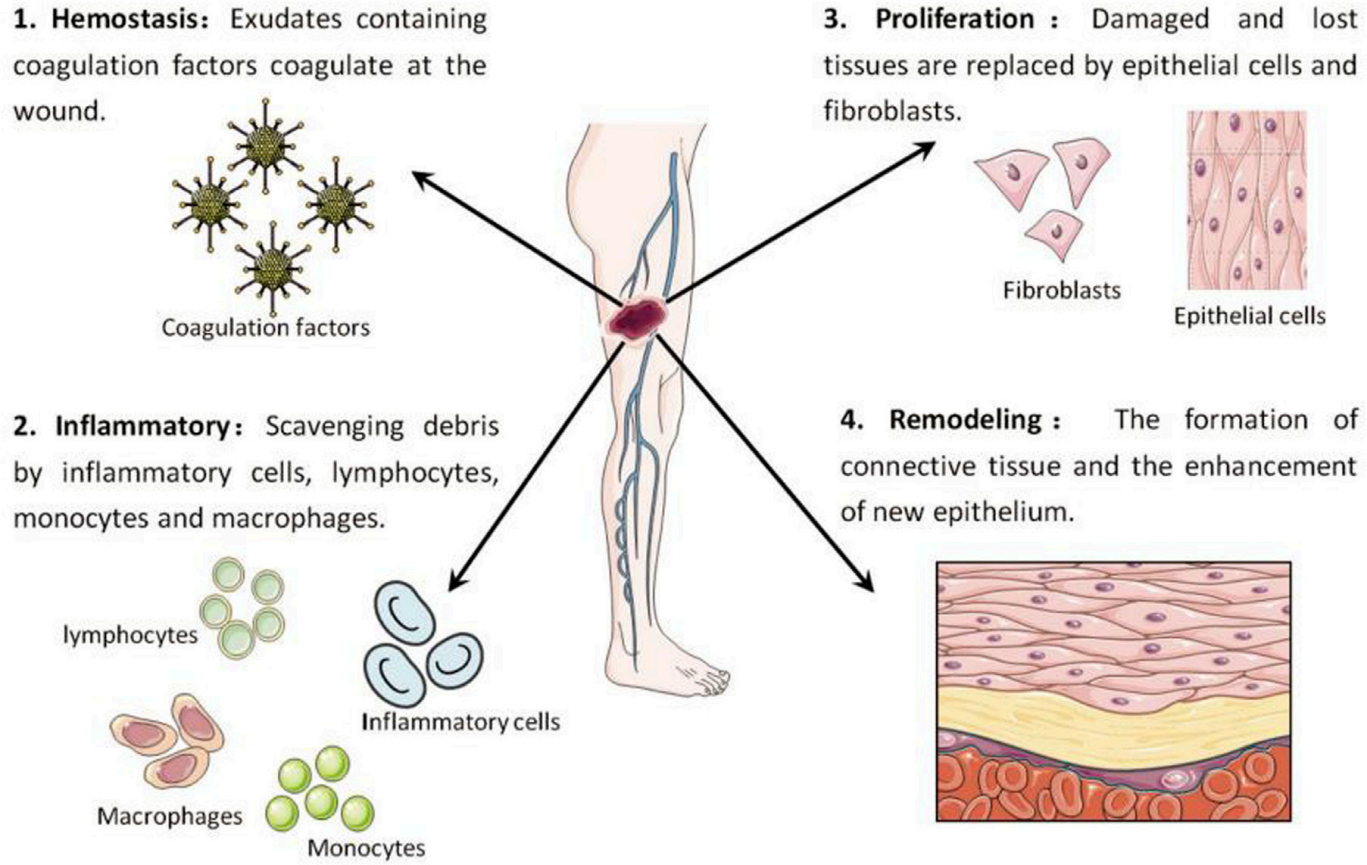
Key stages in the wound healing process: Hemostasis (1), inflammation (2), proliferation (3), and remodelling (4) (Xu et al., 2020).

The process of skin wound healing consists of four separate stages: a) stopping bleeding, b) triggering inflammation, c) promoting cell growth, and d) restructuring (as shown in Figure 1). During the hemostasis stage, the activation of coagulation factors occurs, resulting in the creation of a fibrin clot that offers structural assistance to the damaged tissue. In the phase of inflammation, different types of immune cells, such as inflammation cells, lymphocytes, monocytes, and macrophages, collaborate to eliminate waste from the site of injury. At this stage, neutrophils in the injured area release ROS, antimicrobial peptides, proteolytic enzymes and other substances, and carry out phagocytosis to remove dead cell tissues, foreign bodies and microorganisms in the injured area (Sorg et al., 2017; Raziyeva et al., 2021). At the same time, neutrophils can continuously release pro-inflammatory factors, forming a positive feedback mechanism, prompting more neutrophils to accumulate to the injury site (Hassanshahi et al., 2022). After necrotic tissue and pathogens are removed, neutrophil proliferation is reduced and forced out of the wound. At the same time, the phenotype of macrophages also gradually changed from M1 type, which promoted the release of inflammatory factors, to M2 type, which promoted angiogenesis and tissue regeneration, and wound repair also entered the proliferative stage (Chen C. et al., 2023; Ead and Armstrong, 2023). During the proliferation stage, epithelial cells and fibroblasts undergo active proliferation and migration, gradually filling the exposed wound by forming granulation tissue. During this stage, the combination and placement of components in the extracellular matrix occur, along with the creation of fresh blood vessels. Ultimately, during the maturation or remodelling stage, the granulation tissue is progressively substituted with collagen fibres, leading to the creation of connective tissue (Werner and Grose, 2003; Bainbridge, 2013; Mascharak et al., 2020). It is worth mentioning that these stages do not necessarily occur in separate time periods but instead partially coincide.

In contrast, chronic wounds are wounds that do not heal correctly for a long time, usually without any inclination to regain functional integrity within a 3-month timeframe (Malone-Povolny et al., 2019). The process of healing is not fully complete, with one or more stages, such as hemostasis, inflammation, proliferation, or remodelling, being extended. Studies have indicated that the liquid present in chronic trauma can impede the development of keratinocytes and fibroblasts (Widgerow, 2013). Cell proliferation and migration are severely hindered due to inadequate release of growth factors. Chronic wounds occur when cellular damage and death surpass the ability of the epidermis to grow and regenerate. Chronic illnesses, vascular problems, diabetes, neuropathy, malnourishment, stress, infections, and edema are among the established elements that impact the skin wound recovery procedure (Zhao et al., 2016; Alam et al., 2021).

## 3 The significance and influence of reactive oxygen species in skin regeneration

Wound healing is a natural biological process that occurs when skin tissue is damaged. This process involves the constriction of blood vessels and clot formation, followed by inflammation, cell growth, and finally, the remodelling of the wound. Oxygen is essential in nearly every phase of the wound healing process. This is because energy is needed for various processes involved in wound repair, such as biosynthesis, intracellular transport, and cell motility. ATP synthesis, which acts as the main source of energy, is optimized when oxygen is present. With the process of wound healing, there is an increase in the growth of cells and the generation of the extracellular matrix (ECM), leading to a greater need for energy. Consequently, the demand for oxygen also rises to meet the heightened energy requirements during wound healing (Hunt et al., 1969). Oxygen plays a vital role in the process of wound healing, as it not only supplies energy but also acts as an essential necessity for numerous important enzymatic reactions (Dissemond et al., 2015; Lee et al., 2022).

**FIGURE 2:**
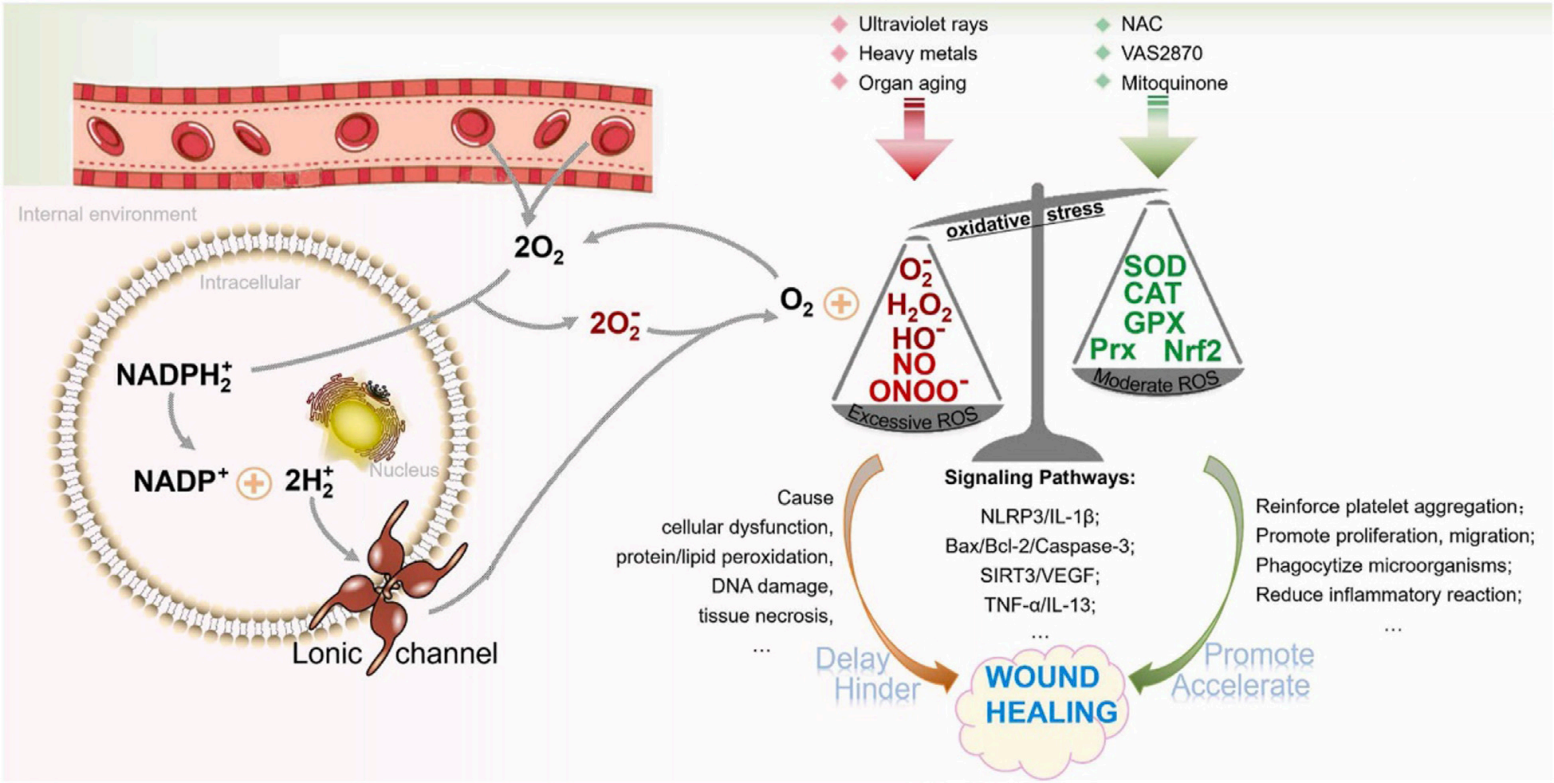
In wound healing, the function of ROS is depicted in figure. Limiting ROS levels to the normal range has a beneficial effect on the wound healing process. Too much reactive oxygen species (ROS) can hinder the cell growth process and cause tissue damage, hindering the wound healing (Wang et al., 2023).

Mitochondria, the endoplasmic reticulum (ER), peroxisomes, various oxidases, and phospholipid metabolism are all potential sources of ROS. In addition, the NADPH oxidase (NOX) family can generate ROS by means of specific enzymes (Bedard and Krause, 2007). Nitrous oxide enzymes are located on cell membranes and can help electrons pass through biofilms, a process that leads to the reduction of oxygen and the production of superoxide (O_2–_). Subsequently, superoxides are able to chemically react to form various reactive oxygen species, such as hydrogen peroxide (H_2_O_2_), peroxyradicals (HO_2–_), and hydroxyl radical. These ROS play diverse roles in cellular functions such as differentiation, proliferation, apoptosis, migration, and contraction (Soneja et al., 2005; Roy et al., 2006). The ROS group includes oxygen derivatives such as hydroxyl radicals (OH·), peroxides, superoxide anions, and hydrogen peroxide (H_2_O_2_). During the non-healing phase of wounds, excessive ROS production occurs, and these ROS molecules oxidize neighboring molecules or cellular components, leading to cellular damage. ROS have been widely regarded as major contributors to cellular damage during the aging process (Beckman and Ames, 1998). Studies have shown that while a moderate amount of reactive oxygen species (ROS) helps maintain intracellular balance, increased ROS levels can have a negative impact on the wound healing process (Zhu et al., 2019). To put it differently, as long as ROS levels remain constant, the integrity and functionality of cells aremaintained. However, ROS also have beneficial effects. The coordinated production of ROS by immune cells is crucial for effective host defense and is essential for cellular signal transduction (Jones et al., 2018; Koo et al., 2019). During the homeostasis phase, ROS produced by NADPH oxidase in blood vessel cells can stimulate chemotactic and adhesion molecule expression, thereby reducing local blood flow (Hoffmann and Griffiths, 2018). During the inflammatory phase, superoxide and H_2_O_2_ produced by neutrophils and macrophages play a crucial role in bacterial killing and preventing wound infection. ROS also has the ability to stimulate tumor necrosis factor-α (TNF-α) and platelet-derived growth factor (PDGF) release and support cell migration (Hoffmann and Griffiths, 2018; Xu et al., 2020; He et al., 2022; Khorsandi et al., 2022). REDOX signaling is also required during the proliferation phase. ROS mediates the tissue growth factor-α 1 (TGF-α1) signaling pathway, improving the expression of fibroblast growth factor (FGF), and promoting the proliferation and migration of fibroblasts and the synthesis and migration of collagen and fibronectin. In addition, ROS can stimulate angiogenesis, endothelial cell division and migration by expressing vascular endothelial growth factor (VEGF), and promote blood vessel formation. Therefore, maintaining an optimal level of reactive oxygen species (ROS) is crucial for fighting against microorganisms and ensuring the survival of cells (as shown in Figure 2).

An increase in ROS at the wound site may lead to the formation of chronic inflammation. Overactivation of ROS can trigger the functioning of transcription factors, such as activator protein 1 (AP-1), mitogen-activated protein kinases (MAPKs), nuclear factor kappa B (NF-κB), and nuclear factor erythroid 2-like 2 (Nrf2) (An et al., 2018; Asai et al., 2018). Nrf2 plays a vital role in protecting against elevated levels of internal oxidative stress and greatly contributes to the healing of wounds (Koike et al., 2020). On the other hand, the stimulation of NF-κB and AP-1 enhances the amount of matrix metalloproteinases (MMPs) in dermal fibroblasts, leading to the degradation of the extracellular matrix (ECM) and a delay in the healing process of wounds. ROS affect the inflammatory reaction in diabetic models by means of the NLR family pyrin domain-containing 3 (NLRP3)-IL-1β pathway (Cheng et al., 2012). Some mechanism studies have also shown that reactive oxygen species ROS hinder the wound recovery process and increase the degree of tissue damage by counteracting the effects of cytokines such as vascular endothelial growth factor (VEGF) and tumor necrosis factor-α (TNF-α) (Lord et al., 2017; Seiwerth et al., 2018).

At present, there is a notable desire to restore ROS levels to enhance the compromised physiological conditions around injuries and facilitate the process of wound recovery. In regards to the problem of excessive accumulation of reactive oxygen species (ROS) near wounds, we provide an overview of the frequently employed materials for ROS removal. These materials are designed to promote cell growth and movement, thereby accelerating the healing process of wounds by decreasing ROS levels within a specific range.

## 4 ROS-scavenging materials

We highlight some natural or synthetic materials for ROS removal and describe their properties in detail. The materials commonly used for ROS removal are shown in Table 2.

**TABLE 2:** Summary of common ROS-scavenging materials.

Category	Representative materials	Description	Reference
Carbon matrix materials	Fullerenes	Fullerenes contains hydroxyl radicals and superoxide anions that can effectively remove ROS, thereby preventing mitochondrial damage and lipid peroxidation	Yin et al. (2009)
	CNTs	CNT is a hollow tubular material composed of carbon atoms, which has excellent electrical conductivity and antioxidant activity, can clear ROS and protect cells from oxidative stress damage	He et al. (2021)
	CQDs	CQDs is a tiny carbon substance that is highly fluorescent and compatible with living organisms, capable of eliminating ROS and reducing oxidative stress	Li et al. (2023)
Metal nanoparticles	Copper nanoparticles	Copper nanoparticles can improve the efficiency of SOD and other enzymes, enhancing the body’s ability to eliminate harmful free radicals	Mondin et al. (2013), Vijayakumar et al. (2019)
	Platinum nanoparticles	Platinum nanoparticles have the ability to promote the conversion of O_2–_ to H_2_O_2_, as well as the ability to promote the conversion of H_2_O_2_ to H_2_O and O_2_. Therefore, it is considered a promising SOD/CAT simulator for the management of oxidative stress	Kim et al. (2010), Yan et al. (2023)
	CeO_2_ NPs	CeO_2_ NPs has significant photolytic and antioxidant properties that promote cell growth and migration while balancing ROS levels in wound	Kim et al. (2020)
Small molecules	TEMPO	TEMPO is composed of nitro radicals such as oxygen and OH, which are able to capture mismatched electrons from other unbound radicals through single-electron transfer of nitrogen oxides	Fink et al. (2007)
Antioxidant enzymes	SOD	SOD is an intrinsic factor that has the ability to clear free radicals and contribute to the differentiation of superoxide radicals, capable of converting them into hydrogen peroxide	Zhang et al. (2021a)
	GPX	GPX can rely on glutathione as an electron donor for enzymatic reduction of H_2_O_2_ and organic peroxide	Brigelius-Flohé and Maiorino (2013)
Natural materials	PDA	The catechol component in PDA has the ability to neutralize free radicals by providing hydrogen atoms of phenolic hydroxyl groups and facilitating reduction reactions by electron transfer	Hu et al. (2020)
	Bilirubin	Bilirubin is an intrinsic antioxidant that effectively inhibits the production of ROS through the NOX pathway and is capable of REDOX sensitive transcription factor hypoxia-Inducible Factor-1α (HIF-1α)	Idelman et al. (2015), Kim et al. (2017)

### 4.1 Carbon matrix materials

After the discovery of fullerenes, a variety of biomaterials made of carbon, such as carbon nanotubes (CNTs), carbon particles, carbon nanoclusters, graphene, graphene quantum dots (GQDs), and carbon quantum dots (CQDs), have been extensively studied and researched (Nash and Ahmed, 2015). Nanomaterials composed of carbon matrices, serving as a novel category of nanomases utilized in biochemical reactions, exhibit enhanced operational durability and reduced expenses compared to natural enzymes. In the meantime, they are easily prepared and exhibit strong resistance to demanding conditions. Notably, fullerenes and their derivatives have demonstrated effective elimination of hydroxyl radicals and superoxide anions. The special physical and chemical properties of carbon-based biomaterials, such as their large specific surface area, good hydrophilicity, and ability to be modified by oxygen-containing functional groups, have attracted great interest and have been widely used in biomedical applications related to ROS.

Fullerenes, also known as buckyballs, are carbon-based molecules that have a spherical shape and possess strong antioxidant properties (Chen X. et al., 2023). These unique structures have the ability to effectively eliminate various ROS, including hydroxyl radicals and superoxide anions. Nanoparticles composed of fullerene have the ability to hinder cytotoxic effects and prevent damage to mitochondria and lipid peroxidation (Yin et al., 2009). Yin et al. (2009) conducted a study where they prepared and assessed three water-soluble variations of fullerenes to determine their effectiveness in eliminating ROS. The three types of fullerenes, specifically C_60_[C(COOH)_2_]_2_, C_60_(OH)_22_, and Gd@C_82_(OH)_22_, exhibited the capacity to safeguard cells against oxidative harm caused by H_2_O_2_, maintain the potential of the mitochondrial membrane, and hinder the production of intracellular ROS. Furthermore, the clearance efficiency of ROS was assessed for the three substances, where Gd@C_82_(OH)_22_ demonstrated the most superior effectiveness, trailed by C_60_(OH)_22_ and C_60_[C(COOH)_2_]_2_. *In vitro*, these compounds were also discovered to efficiently eliminate stable DPPH (2,2-diphenyl-1-picrylhydrazyl) radicals and ROS and hinder lipid peroxidation. The results indicated that fullerene derivatives have the potential to be used as protectants and treatments for cells in living organisms.

There are also other carbon matrix materials, such as carbon nanotubes (CNTs). CNTs are hollow tubular structures formed from carbon atoms that have excellent electrical conductivity and mechanical strength. Some types of CNTs show good antioxidant activity, clearing ROS and protecting cells from oxidative stress (He et al., 2021). Graphene is a carbon-based substance consisting of a solitary layer of carbon atoms, possessing exceptional electrical conductivity and chemical stability. Graphene and its derivatives exhibit remarkable capability in scavenging ROS and can effectively neutralize various types of ROS (Tang et al., 2019). CQDs are tiny carbon substances that exhibit strong fluorescence and are compatible with living organisms. Some specific CQDs possess antioxidant characteristics and are capable of eliminating ROS and diminishing oxidative stress (Li et al., 2023). Black phosphorus is a carbon material at the nanoscale that possesses a significant surface area and strong adsorption capability. As an antioxidant, it has the ability to absorb and counteract ROS (Huang et al., 2022). Various Carbon matrix materials have different mechanisms and characteristics in eliminating ROS. In the biomedical domain, these materials are extensively researched and employed for the purpose of controlling oxidative stress and safeguarding cells against oxidative harm.

### 4.2 Metal nanoparticles

The research on the elimination of reactive oxygen species in metal-containing biomaterials has made substantial progress because of the discovery of gold nanoparticles with the function of removing ROS, as well as Fe_3_O_4_ nanoparticles with peroxidase activity (Ju et al., 2023; Xi et al., 2023). Metal-based materials are a class of inorganic antioxidants with strong ROS scavenging ability. The biomaterials made of metal have catalytic characteristics that resemble peroxidase (POD), catalyst (CAT), superoxide dismutase (SOD), glucose oxidase (GOx), and glutathione peroxidase (GPx), allowing them to effectively eliminate ROS.

**FIGURE 3:**
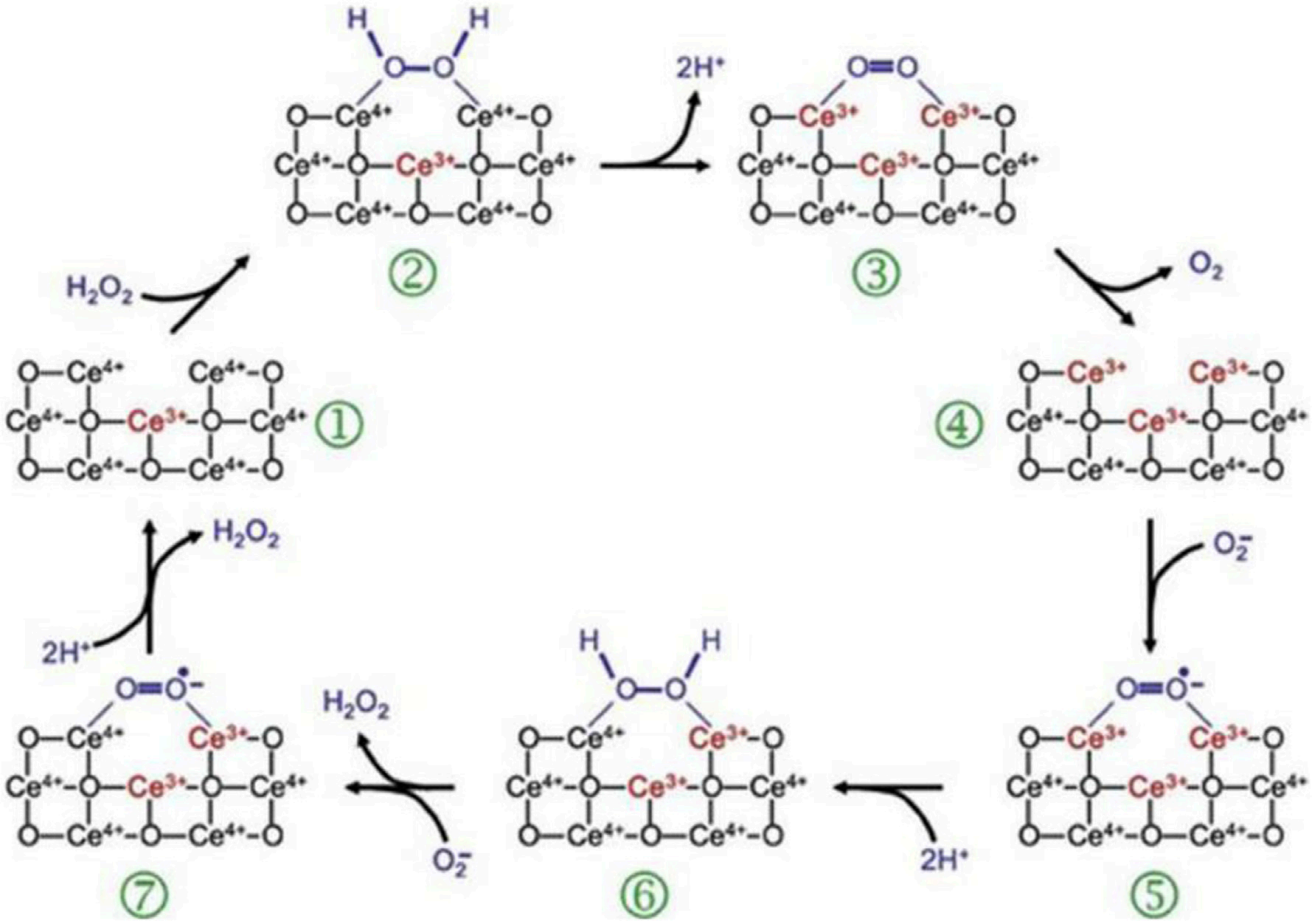
ROS-scavenging mechanism of CeO_2_ NPs (Celardo et al., 2011).

CeO_2_ NPs, known as cerium nanoparticles, possess significant photolytic and antioxidant characteristics, which make them a potential therapeutic option for a range of ailments, including wound healing and other medical conditions (Wang et al., 2020; Xu et al., 2023). Earlier research on CeO_2_ has discovered its ability to enhance the growth and migration of cells while also balancing the levels of ROS in chronic ulcer, consequently accelerating the process of wound healing (Xu et al., 2023). Bhang et al. prepared a Cu-deposited cerium oxide nanoparticle (CuCe NPs), which showed strong antioxidant effect, significantly promoted the anti-inflammatory effect and M2 polarization of macrophages, and alleviated tissue damage (Im et al., 2023). Furthermore, CeO_2_ nanoparticles have the capability to imitate the antioxidant function of superoxide dismutase (SOD) and catalase (CAT), potentially expediting the process of wound healing (Rather et al., 2018). The mixed valence states of Ce_3_ and Ce_4_ are responsible for the antioxidant characteristics of cerium oxide nanoparticles. Cerium oxide nanoparticles have become widely utilized inorganic nanomaterials due to their exceptional antioxidant characteristics and capacity for automatic regeneration (Huang et al., 2021) (Figure 3).

Platinum (Pt) nanoparticles are frequently employed as catalysts in synthetic chemistry for hydrogenation and oxidation reactions. Additionally, Pt is an inherent element of cisplatin and other chemotherapy drugs. Research has indicated that Pt has the ability to facilitate the transformation of O_2–_ into H_2_O_2_, as well as the transformation of H_2_O_2_ into H_2_O and O_2_ (Umeda et al., 2011; Yan et al., 2023). Consequently, it is regarded as a promising SOD/CAT simulator for managing oxidative stress. Kim et al. (2010) connected Pt with the HIV-1 TAT fusion protein and found that Pt can improves cell endocytosis, produces antioxidant effects, and has higher bioavailability and lower toxicity. Moreover, studies have shown that TAT-Pt nanoparticles (TAT-Pt NPs) can increase the nematode survival rate under acute oxidative stress induced by paraquat, as well as in the presence of chronic endogenous ROS. These results suggest that TAT-Pt nanoparticles have the potential to be an effective drug for the treatment of chronic inflammation.

Copper nanoparticles, found in superoxide dismutase and glutathione, boost the effectiveness of SOD and other enzymes, enhancing the body’s capacity to eliminate harmful free radicals. Copper impacts distinct signaling pathways in living organisms, such as the nuclear factor E2-associated factor (Nrf) signaling pathway and the mitochondrial signaling pathway mediated by CDK4. By means of this phenomenon, the body can maintain homeostasis and avert inflammatory ailments (Iskra and Majewski, 2000; Wang et al., 2015).

Moreover, various inorganic nanoparticles, such as gold, selenium, and vanadium, demonstrate impressive abilities to scavenge ROS. The metal nanoparticles exhibit the capacity to efficiently combat environments with oxidative stress and contribute to safeguarding cells (Vernekar et al., 2014; Rosenkrans et al., 2020). However, the use of metal-based biomaterials for ROS removal in the clinic is limited due to poor blood solubility, lack of stability, and the risk of unknown toxicity *in vivo* over a long period of time.

### 4.3 Small antioxidant molecules

Natural organic small molecule ROS scavengers can be extracted from natural sources such as fruits, vegetables, herbs, tea leaves, and dietary supplements. The FDA has approved certain natural compounds for the treatment of diseases associated with oxidative stress. These compounds include catechins, baicalin, vitamins A, C, and E, coenzyme Q10 (CoQ_10_), and N-acetylcysteine (NAC) (Firuzi et al., 2011; Mut-Salud et al., 2016). In addition, many artificial organic small molecule reactive oxygen scavengers have also been applied to remove reactive oxygen species. For example, butylated hydroxyanisole (BHA), butylated hydroxytoluene (BHT), and their analogues are used as synthetic ROS scavengers (Dassarma et al., 2018). Nevertheless, the utilization of these artificial substances is restricted because of the potential dangers they may present, such as liver toxicity, kidney toxicity, and carcinogenic risks.

TEMPO is a widely recognized reactive oxygen species (ROS) scavenger consisting of oxygen and nitro radicals such as OH and O_2_ (Fink et al., 2007; Nag et al., 2022). TEMPO seizes unmatched electrons from other unbound radicals via nitrogen oxide’s single-electron transfer, causing the redox reaction to alternate between the oxidized form of nitrogen oxide, oxammonium cation, and hydroxylamine (Soule et al., 2007). Hence, scientists have been committed to creating polymer nanomaterials with antioxidant properties by incorporating TEMPO or its derivatives into the polymer structure and utilizing them for the management of diverse ailments (Yamada et al., 2003; Marciniak et al., 2016).

### 4.4 Antioxidant enzymes

Several natural enzymes, such as superoxide dismutase (SOD), catalase (CAT), and glutathione peroxidase (GPx), have the capacity to remove ROS. These enzymes can facilitate the breakdown of ROS, thereby reducing oxidative damage. Superoxide dismutase (SOD) is a vital enzyme with antioxidant properties that are essential for combating oxidative stress. SOD, as an intrinsic factor, has the ability to clear free radicals, and contributes to the differentiation of superoxide free radicals, converting them into hydrogen peroxide (Zhang W. Q. et al., 2021). Glutathione peroxidase (GPx) enzymatically reduces H_2_O_2_ and organic peroxides, relying on glutathione as an electron donor. Humans possess a total of eight GPx genes, namely, GPX1-8. Among these, GPX1-4 are selenoproteins that include selenocysteine (SeCys) residues within their catalytic core, as stated in reference (Brigelius-Flohé and Maiorino, 2013). Wound injuries frequently show reduced levels of glutathione and GPx activity because GPx relies on glutathione as an electron provider. Despite the reduced levels of GPX1 protein in damaged skin, GPX1 mRNA expression is increased in wound injuries, and both protein and GPx levels are decreased in normal rat wounds (Steiling et al., 1999; Iuchi et al., 2010).

### 4.5 Natural materials

Bilirubin is a metabolite naturally present in the human body, which is both an intrinsic antioxidant and a molecule involved in regulation. Under conditions of oxidative stress, insoluble bilirubin undergoes oxidation to form soluble biliverdin. Bilirubin can effectively inhibits the production of ROS through the NOX pathway and inhibits lipopolysaccharide (LPS) -mediated inflammation by targeting the REDOX sensitive transcription factor hypoxia-Inducible Factor-1α (HIF-1α) (Idelman et al., 2015). To enhance the antioxidant properties of bilirubin, Jon et al. utilized covalent bonding and self-assembly methods to create nanoparticles consisting of bilirubin conjugated with polyethylene glycol (PEG-BR NPs) (Kim et al., 2017). PEG-BR nanoparticles not only maintain the antioxidant capacity of natural bilirubin, but also show greater stability and durability in living organisms. Moreover, they promote the polarization of M1 macrophages towards the M2 phenotype through the inhibition of inflammatory cytokine secretion, thereby alleviating inflammation. In summary, because of its beneficial properties as an antioxidant and anti-inflammatory agent, bilirubin presents a promising approach for treating inflammatory conditions.

Polydopamine (PDA) is a melanin-like substance that has antioxidant properties. At present, researchers have found that PDA may have the effect of scavenging free radicals due to the REDOX chemistry of its polyphenol structure, as well as endogenous free radical tolerance and effective energy transfer properties (Hu et al., 2020). The catechol component has the ability to neutralize free radicals by providing hydrogen atoms to the phenol hydroxyl group and promoting a reduction reaction by electron transfer. The interaction between the produced phenoxyl radicals and secondary quenching radicals results in the creation of enduring quinone structures. PDA is often utilized to scavenge ROS in the treatment of conditions resulting from oxidative stress. PDA NPs are renowned for their ability to scavenge free radicals and can be employed to counteract different forms of ROS. PDA provide a convenient and efficient method for creating coatings on various material surfaces. These coatings possess a wide range of impressive biological characteristics, such as anti-inflammatory, anticancer, and antioxidant properties (Dai et al., 2019). When using natural materials as ROS scavengers *in vivo*, the biocompatibility and biodegradability of the materials should be considered. It is very important to take inspiration from natural materials that have an innate ability to remove ROS, as this will greatly facilitate the creation and development of ROS-scavenging materials.

## 5 Application of ROS-scavenging materials in wound healing

**FIGURE 4:**
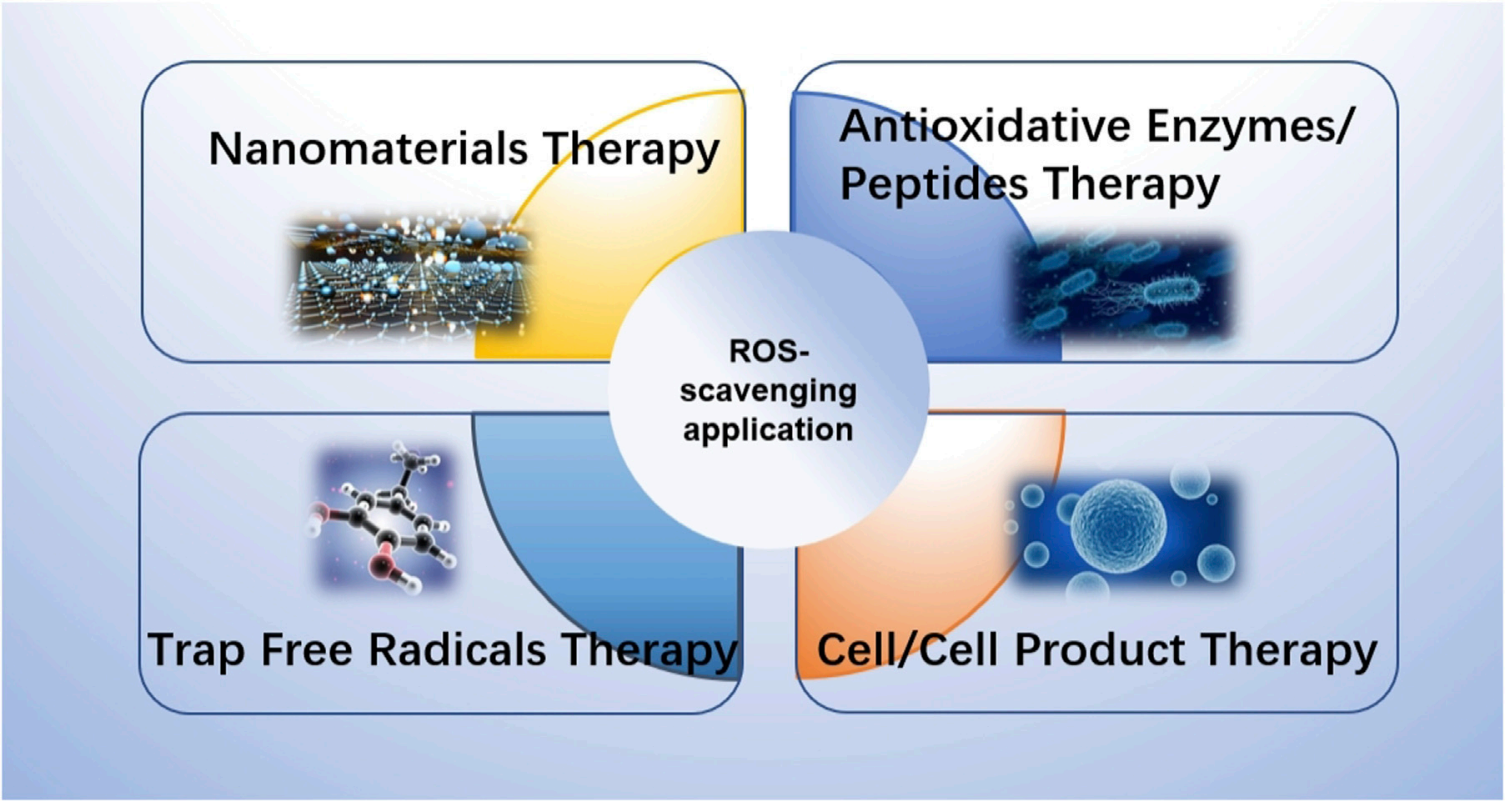
Schematic diagram of the application of ROS scavenging materials in skin wound repair.

ROS scavenging materials have shown the ability to effectively regulate ROS levels during injury, manage oxidative stress, and promote wound healing. Over the past few years, many scientists have studied different substances with antioxidant properties to promote the process of wound healing. These materials possess diverse characteristics and functions, enabling them to promote wound healing through various mechanisms, such as enhancing cell proliferation and migration, modulating inflammatory responses, facilitating angiogenesis, and promoting collagen synthesis. In this chapter, we will discuss the application of these materials in skin wound repair (Figure 4).

### 5.1 Utilizing nanomaterials for the purpose of promoting wound healing

In the context of skin wound healing, the application of nanomaterials has shown great potential in promoting wound healing through multiple mechanisms. Nanomaterials possess a large surface area-to-volume ratio and highly tunable physical properties, which can enhance cell adhesion and migration, thereby promoting epithelial cell healing and regeneration at the wound site. In this subsection, we will primarily focus on discussing the applications of nanomaterials in facilitating skin wound healing. We will introduce different types of nanomaterials, including metallic nanoparticles and nonmetallic nanoparticles, and provide detailed descriptions of their mechanisms in enhancing cell adhesion and migration, modulating inflammatory responses, promoting angiogenesis, and facilitating collagen synthesis.

According to Rather et al. (2018), CeNPs actively combat cellular oxidative harm by efficiently eliminating ROS. In some studies, polycaprolactone (PCL)-gelatin nanofibers (PGNPNF) were prepared by electrospinning technology, and their antioxidant capacity was tested. The XRD analysis indicated a reduction in crystallinity of approximately 2.6 times when compared to the original PCL, which facilitated the quick degradation of nanofibers and the subsequent release of CeNPs. PGNPNF meshes exhibited superoxide dismutase (SOD)-mimetic activity. The proliferation of 3T3-L1 cells on PGNPNF meshes was confirmed to have increased by approximately 48% according to SEM analysis. Ma et al. employed electrostatic interactions to develop a biocompatible nanocomposite called MoS_2_−CeO_2_, formed by combining PEG-MoS_2_ and CeO_2_ (Ma et al., 2021). Upon histological examination of tissue samples from the nanocomposite and near-infrared laser-treated groups, it was observed that inflammation was decreased, fibroblast migration was enhanced, and collagen fibres were densely arranged in a parallel manner. This was in stark contrast to the control group, where collagen deposition appeared uneven. The control group showed lower collagen synthesis than all treatment groups.

**FIGURE 5:**
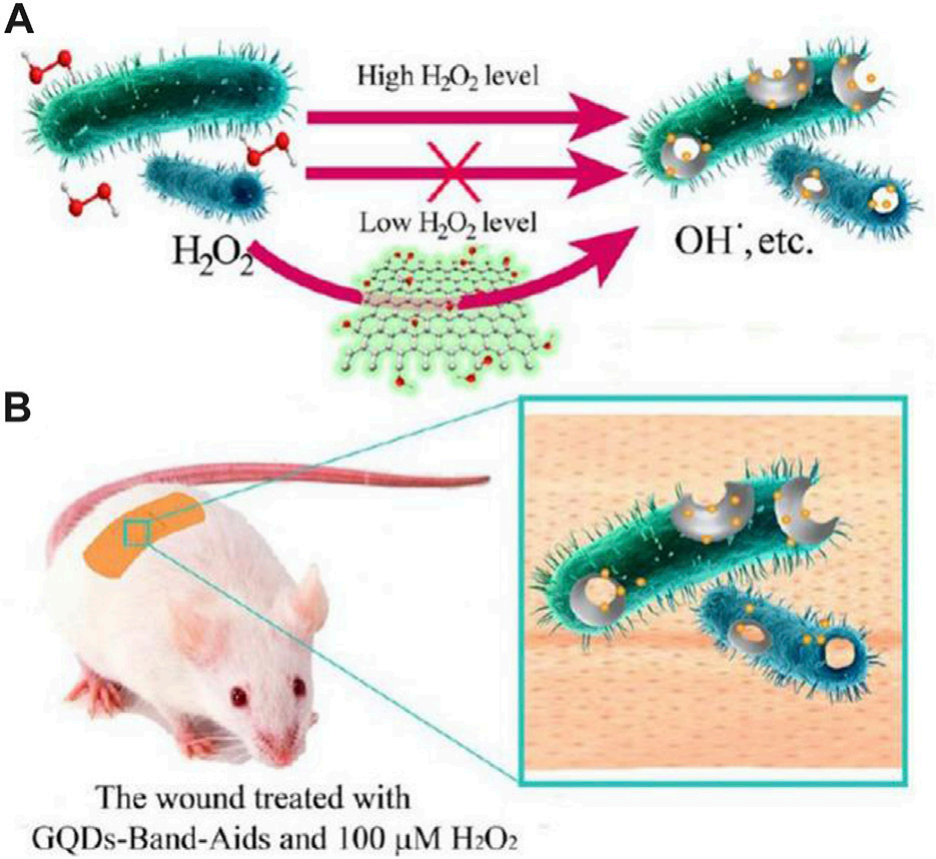
**(A)** The designed system based on GQDs and low level of H_2_O_2_ for the antibacterial application. **(B)** The GQD-Band-Aids used in wound disinfection *in vivo* (Sun et al., 2014).

In summary, MOS_2_-CEO_2_ nanocomposites combined with near-infrared 808 nm laser therapy exhibit significant antibacterial, antioxidant, anti-inflammatory and regenerative properties, ultimately promoting rapid healing of chronic skin injuries. Sun and his colleagues synthesized graphene quantum dots (GQDs) materials and found that GQDs exhibit peroxisase-like properties because they are able to help break down H_2_O_2_ and have strong antibacterial effects (Sun et al., 2014). In all *in vitro* studies, GQDs showed strong inhibitory effects against both gram-negative bacteria (*E. coli*) and Gram-positive bacteria (*Staphylococcus aureus*). By utilizing these characteristics, band-aids based on GQDs were created, showcasing remarkable antibacterial efficacy in living organisms (Figure 5). Using a similar strategy, hybrid nanozymes consisting of Au-g-C_3_N_4_ were synthesized for antibacterial applications (Wang et al., 2017). The hybrid nanozymes displayed enhanced peroxidase-like activity due to the synergistic effect of combining Au nanoparticles with C_3_N_4_ sheets. Moreover, this collaborative impact enabled a decrease in the necessary amount of H_2_O_2_ for antibacterial uses. *In vitro*, the research displayed impressive bactericidal effects on both gram-negative and gram-positive bacteria. In addition, it effectively degrades existing bacterial biofilms and hinders the formation of new ones.

Li et al. (2021) prepared a hybrid hydrogel (β-GO/RB/PVA HD) consisting of oxidized graphene, rose bengal, and polyvinyl alcohol. A comprehensive assessment was conducted on the mechanical characteristics and antibacterial effects activated by light from the hydrogel. The β-GO inorganic mesh intertwined with the porous framework of PVA, leading to a notable enhancement in the hydrogel’s mechanical characteristics. Both *in vitro* and *in vivo* experiments have shown that the heat generated by β-GO under 808 nm light irradiation and the ROS generated by RB under 550 nm light irradiation can produce significant antibacterial effects in as little as 10 min. In addition, the mixed hydrogels significantly accelerated the healing of infected wounds.

In the same way, substances such as platinum, copper and Prussian blue are also highly antioxidant due to their ability to mimic natural antioxidant enzymes such as catalase and superoxide dismutase (SOD). The utilization of these carriers can be employed to investigate wound healing activities. In addition, antioxidant nanomaterials can also serve as metal-organic frameworks (MOFs), where the highly permeable arrangement of MOFs allows unpaired electrons to interact with active sites within the material, enhancing the catalytic performance of these unpaired electrons (Cheng et al., 2017).

### 5.2 Skin wound therapeutic application of enzymes and peptides with antioxidant properties

As stated in the preceding section, superoxide dismutase (SOD) is an antioxidant enzyme that plays a role in combating oxidative stress. In the process of chronic wound healing, insufficient synthesis of superoxide dismutase (SOD) will aggravate oxidative stress and hinder effective wound healing. Research has shown that the use of hydrogels containing SOD significantly improves the recovery of diabetic ulcers (Zhang et al., 2018). Similarly, in a mouse burn model, plasma thiobarbituric acid reactivity (TBAR) metabolite levels were significantly reduced by intravenous administration of 10 mg/kg Cu/Zn-SOD[poly (butyl ester) -coupled SOD] (Fitzmaurice et al., 2011). Compared to the control rats, the animals treated with SOD exhibited a 7-day improvement in survival.

**FIGURE 6:**
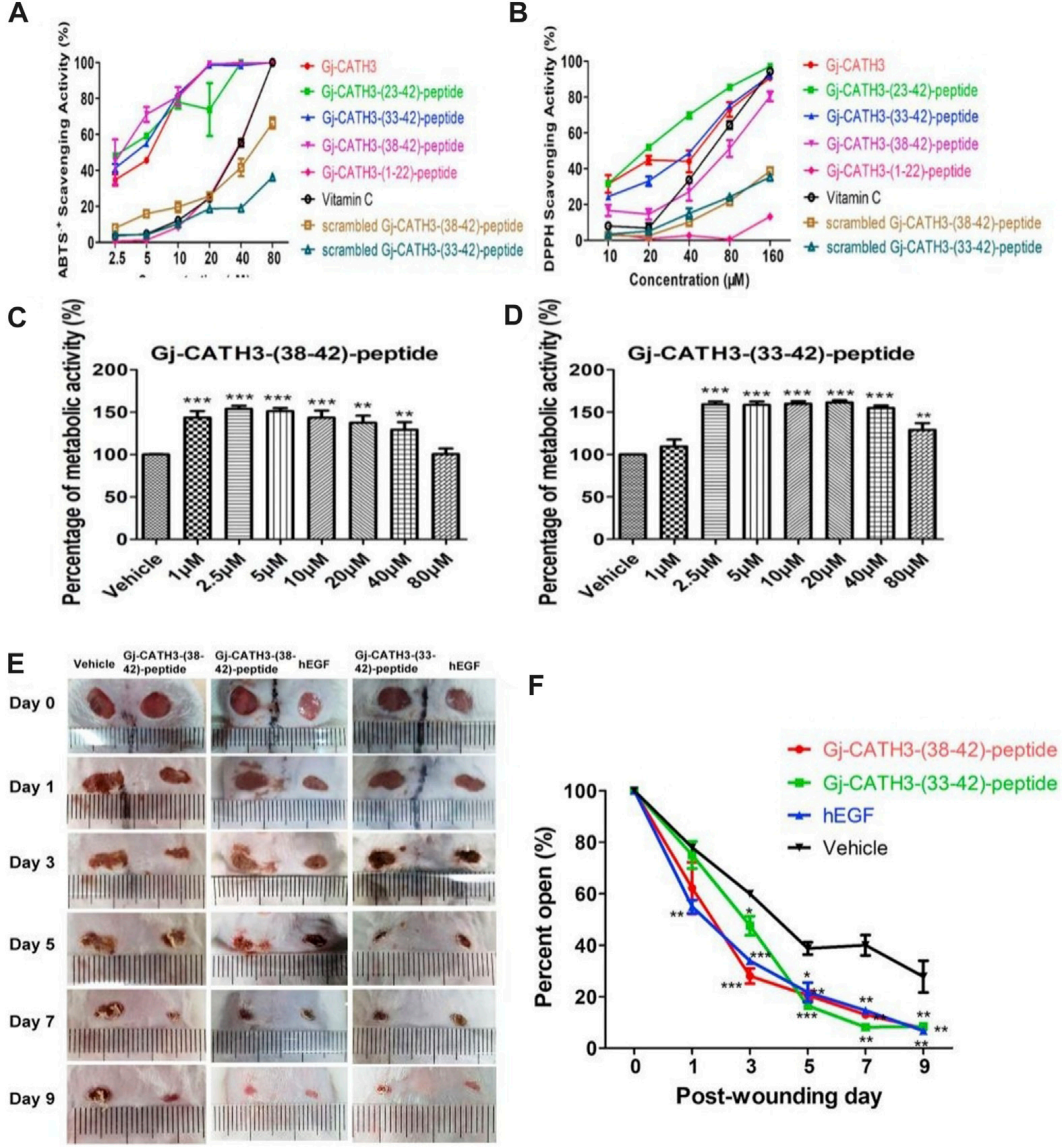
Biocompatibility and antioxidant activity of Gj-CATH3 peptide analogs *in vivo* and *in vitro*. **(A)** ABTS + free radical and **(B)** DPPH free radical scavenging activity of Gj-CATH3 peptide analogs. **(C,D)** Effect of Gj-CATH3 peptide analogs on the proliferation of HaCaT cells. *In vivo* assessment of the Gj-CATH3 peptide analogs for wound healing **(E)** Photographic snapshots of temporal development of healing wounds for the different Gj-CATH3 peptide analogs in 0–9 days. **(F)** Wound closure rate of different Gj-CATH3 peptide analogs at different healing times (Cai et al., 2021).

Antioxidants and antimicrobial peptides are also commonly used in the treatment of skin wounds, and their effects include fighting oxidative substances, regulating the production of cytokines, aiding cell movement and growth, and promoting the formation of new blood vessels (Steinstraesser et al., 2008). Cai et al. (2021) improved cell sensitivity by constructing Gj-CATH3 peptide analogues. Gj-CATH3 analogues showed high antioxidant activity. However, experiments on cytotoxicity and hemolysis demonstrated a notable decrease in toxicity. Additional *in vitro* investigations conducted on HaCaT cells revealed a noteworthy enhancement in cellular growth. Preclinical experiments additionally demonstrated impressive effectiveness in healing wounds, along with a significant increase in the activity of superoxide dismutase (SOD) (Figure 6). Calycosin-7-glucoside (CG) is an isoflavone component found in Astragalus membranaceus (AR), commonly known as Huangqi. Chen J. et al. (2023) investigated the mechanism of CG in treating diabetic wounds by using a diabetic wound model and an LPS- and IFN-γ-induced inflammation model in RAW264.7 cells. The study results showed that CG accelerated wound healing and promoted granulation tissue regeneration in diabetic mice. Protein chip technology revealed that CG increased the production of M-CSF, G-CSF, GM-CSF, IL-10, IL-13, and IL-4 but did not increase MCP-1, IL-1β, IL-1α, TNF-α, and TNF-RII. Furthermore, CG increased the proportion of anti-inflammatory Ly6CLo/- monocytes in peripheral blood and M2 macrophages in the wound. ELISA and flow cytometry analysis showed that CG enhanced the expression levels of IL-10, VEGF, CD206, and Arg-1 while significantly reducing the levels of IL-1, IL-6, IL-12, TNF-α, CD86, and iNOS. CG promoted the recruitment of anti-inflammatory monocytes and reduced the mitochondrial glycolysis rate, thus inducing M2 macrophage polarization through the ROS/AMPK/STAT6 pathway. These findings suggest that CG may be a promising therapeutic agent for diabetic wound treatment. According to Cao et al. (2018), cathelicidin-OA1 from Odorrana andersonii, which is a cathelicidin known for its strong antioxidant properties, was discovered. Cell proliferation in HaCaT and fibroblasts was observed *in vitro*, and it was found to be dependent on both the dose and time. The histopathological findings on live tissues during the initial phases of wound healing revealed improved tissue re-epithelialization and the formation of granulation. The experimental rats showed greater wound-healing ability than the other rats. In addition, elevated levels of TNF-α and TGF-β were observed in the experimental group, which can cause growth factors and cells to be attracted to the injury site, ultimately enhancing the wound healing rate.

Hussain et al. (2021) created a dopamine-modified multidomain peptide (DAP) that possesses numerous advantageous characteristics for the process of wound healing. The attributes encompass the ability to combat microbes, the capacity to scavenge reactive oxygen species (ROS), and the ability to adhere. Cell proliferation was significantly increased by treating 3T3 fibroblasts with DAP. In the mouse full-thickness model, the immunohistochemical analysis revealed a notable decrease in pro-inflammatory indicators such as IL-6 and TNF-α among the rats treated with DAP. Rats treated with DAP exhibited significant growth of blood vessels and accumulation of collagen. In the study conducted by Zhang J. et al. (2021), a new antioxidant peptide was obtained from blackfish. Among the four tested peptides, SDGSNIHFPN and PGLMLGGSPPGLLGGSPP exhibited the most pronounced antioxidant properties. The studies on cell viability showed that they have the ability to shield cells from damage caused by H_2_O_2_. Additionally, the investigation revealed that these peptides not only functioned as powerful antioxidants through the activation of the KEAP1-Nrf2 signaling pathway but also displayed promise as a therapeutic approach to enhance the speed of wound healing.

### 5.3 Utilize free radical capture treatment for the healing of wounds

Free radical capture therapy effectively reduces ROS levels by introducing materials with antioxidant properties, thereby reducing oxidative stress and the inflammatory response and promoting the wound healing process. These ROS-scavenging materials can work through a variety of mechanisms. First, they can react directly with ROS, neutralizing harmful free radicals and thereby reducing tissue damage and inflammation. Second, they can also regulate the balance of ROS production, prevent excessive ROS production, maintain redox balance, and facilitate normal cell function and wound healing. In this chapter, we will focus on the use of free radical capture therapy to promote skin wound healing. Various natural enzymes, including glutathione (GSH) peroxidase, superoxide dismutase (SOD), and catalase, have been discovered to possess the capability of diminishing levels of free radicals. Free radical scavengers interrupt the peroxide chain by interacting with free radicals. Nevertheless, in the presence of a substantial quantity of unbound radicals (e.g., O_2–_), this mechanism will be impeded. Therefore, as the disease progresses, the body experiences the accumulation of free radicals that hinder the action of these enzymes. Therefore, the solution to this dilemma must use antioxidants.

Due to their identical enzymatic catalytic activity to SOD, PDA NPs exhibit enhanced stability and durability. It is extensively utilized in the management of ailments resulting from oxidative stress. Jing et al. (2020) developed 2D nanosheets (NSs) composed of polydopamine (PDA), referred to as PDA NSs. PDA NSs has the ability to scavenge free radicals such as O_2–_, ABTS and DPPH. In addition, PDA NSs showed significant antioxidant activity against O_2_ free radicals. Furthermore, PDA NSs exhibited anti-inflammatory characteristics. *In vivo* experiment results showed that on the 14th day of administration of 60 μg/mL experimental animals, the wound was completely healed without obvious scar formation. The histological assessment of the treatment group revealed a notable reduction in inflammatory cells and an increased level of collagen accumulation at the site of the wound in comparison to the control group. Fu Y. et al. (2021) developed a plan to improve the antioxidant capability of polydopamine nanoparticles (PDA NPs) through the creation of reduced PDA NPs. These reduced PDA NPs exhibit potent antioxidant properties and are incorporated into a hydrogel dressing to prevent cellular stimulation caused by external oxidative stress. As a result, this approach promotes wound healing rate.

**FIGURE 7:**
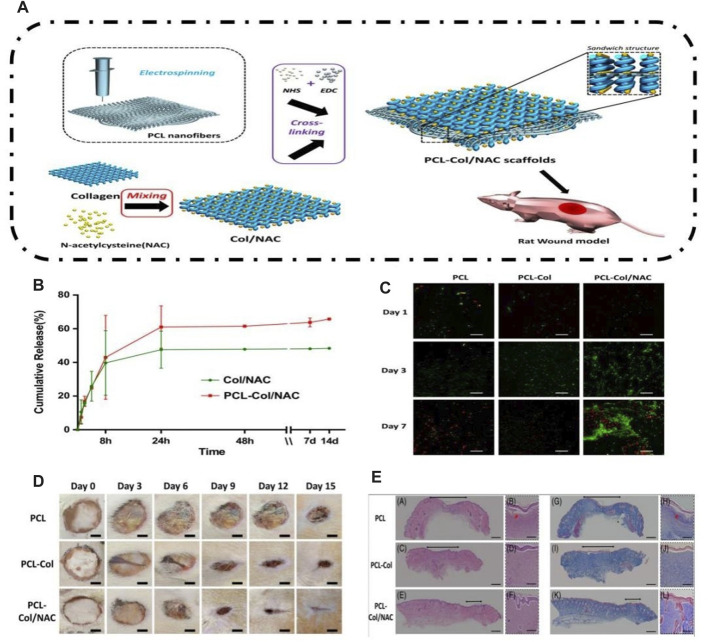
Preparation of the PCL-COL/NAC scaffold and its effect on skin tissue regeneration. **(A)** Schematic of experimental procedures for fabricating the PCL-COL/NAC scaffold. **(B)** Time-dependent cumulative release profiles of NAC by Col/NAC and PCL-COL/NAC. **(C)** Confocal fluorescence images for FDA/PI staining of NIH 3T3 fibroblasts cultured on the scaffolds for 1, 3, and 7 days. **(D)** Photographic snapshots of the temporal development of healing wounds for the different scaffolds at 0 and -15 days. **(E)** H&E staining and Masson staining images of the wound section on the 15th day for each group (Hou et al., 2019).

To meet the requirements of wound healing, N-acetylcysteine (NAC) acts as an antioxidant by neutralizing excessive production of ROS and promoting both angiogenesis and epidermal maturation. Hou et al. (2019) utilized a molding method to create a sandwiched framework scaffold, known as the PCL-COL/NAC scaffold. This scaffold comprised a core of PCL nanofibers surrounded by collagen loaded with NAC on both sides (Figure 7). The performance of the PCL-Col/NAC scaffold is outstanding, and it consistently delivers drugs over time. The *in vitro* experiments demonstrated that PCL-Col/NAC scaffolds exhibited superior attributes in terms of cell migration and proliferation compared to the control group. After 21 days, the assessment of the complete skin defect wound model demonstrated a positive treatment result and enhanced development of the outer layer of the skin when compared to the control group. CD31 staining in immunohistological analysis revealed a comparatively greater abundance of recently developed blood vessels in the PCL-Col/NAC group than in the control group.

### 5.4 Using cells and cell products for the purpose of wound healing

Stem cells, exosomes, and cytokines have emerged as promising therapeutic strategies for promoting skin wound healing. Stem cells are a category of cells with self-renewal and multilineage differentiation capabilities (Fu X. et al., 2021). They can differentiate into various cell types, including epithelial cells, endothelial cells, and fibroblasts, thereby facilitating wound healing at different stages. Exosomes, on the other hand, are small vesicles secreted by cells and are rich in bioactive molecules such as proteins, nucleic acids, and growth factors (Yang et al., 2023). They possess regulatory functions similar to their parent cells and can promote wound healing by delivering these bioactive molecules. Additionally, cytokines play a crucial role in wound healing (Behm et al., 2012). They are signaling proteins produced by cells that can regulate various aspects of wound healing, including cell proliferation, migration, and differentiation, as well as inflammation modulation and angiogenesis. Although some studies have reported the application of stem cells, exosomes, and cytokines in promoting skin wound healing, their specific mechanisms and effects require further investigation. This chapter aims to provide an overview of the applications of stem cells, exosomes, and cytokines in promoting skin wound healing and explore their role in ROS clearance. We will review relevant research findings, including the impact of these therapeutic strategies on wound healing speed, epithelial regeneration, angiogenesis, and inflammation modulation. Furthermore, we discuss the mechanisms by which stem cells, exosomes, and cytokines clear ROS, as well as their potential clinical applications in promoting skin wound healing.

MSCs promote the movement and growth of important cells by combining fibronectin and collagen proteins while also controlling proteins associated with the extracellular matrix (Chen et al., 2012). There has been an increasing fascination in the potential of MSCs derived from bone marrow in the realm of wound healing therapy. Mohanty and Pradhan created a biomaterial bandage called EGF-curcumin and incorporated it into MSCs, resulting in the formation of MSCs-EGF-Cur B (Mohanty and Pradhan, 2020). The application of EGF-Cur B to MSCs led to a significant increase in the expression of MSC transcription factors, exceeding the levels observed in MSCs cultured using conventional methods. EGF-Cur B demonstrated superior effectiveness in enhancing cell proliferation compared to curcumin B alone. In rats, the administration of MSCs-EGF-Cur B greatly improved the healing of wounds, the production of collagen proteins, the formation of granulation tissue, and the growth of new blood vessels. Wu et al. (2007) and de Mayo et al. (2017) investigated the effects of mesenchymal stem cells (MSCs) on skin wound healing using bone marrow-derived MSCs (BM-MSCs). Luo et al. (2010) examined the effects of umbilical cord blood-derived MSCs (UCB-MSCs). These studies yielded positive results both *in vitro* and *in vivo*, demonstrating that stem cell therapy can accelerate wound healing through direct promotion of skin regeneration by expressing keratin and forming glandular structures, promoting the formation of blood vessel-like structures by endothelial cells, reducing inflammation, and decreasing collagen deposition. Researchers have also investigated whether adipose tissue-derived MSCs from patients with chronic kidney disease (CKD) have the same functionality as cells from normal sources in wound healing (Khanh et al., 2017). It was found that uremic toxins induced increased expression of ROS in na-MSCs, leading to decreased expression of hypoxia-inducible factor-1α (HIF-1α) under hypoxic conditions. CKD-AT-MSCs isolated from early-stage CKD patients exhibited a significant imbalance in redox state and high expression of ROS. In a mouse skin flap model, nAT-MSCs promoted inflammatory cell recruitment and ischemic recovery, while CKD-AT-MSCs showed functional defects and delayed wound healing processes. Importantly, the expression of prolyl hydroxylase domain 2 (PHD2) was selectively increased in CKD-AT-MSCs, and its inhibition restored HIF-1α expression and the wound healing function of CKD-AT-MSCs. These findings suggest that more research is needed on the functionality of bone marrow-derived mesenchymal stem cells in CKD patients before their clinical application.

The biological effects of MSC-derived extracellular vesicles (EVs) are recognized to be similar to those of MSCs in terms of tissue regeneration, maintenance of homeostasis, and wound healing (Ding et al., 2023). Shiekh et al. (2020a) developed a specialized wound dressing called OxOBand, which incorporates extracellular vesicles (EVs) loaded with oxygen-releasing antioxidants. This innovative dressing was specifically designed to assist in the closure of chronic wounds and promote skin regeneration. According to the research, the EVs utilized in OxOB promoted the migration of human fibroblasts and keratinocytes while also enabling fibroblasts and keratinocytes to have an extended lifespan in situations of elevated glucose levels. Following a 2-week duration, a significant reduction in oxidative stress was noted in diabetic wounds that were subjected to the OxOB and dressing, in contrast to diabetic control wounds. Moreover, the utilization of the OxOBand dressing promoted the proliferation of epithelial cells and hair follicles.

**FIGURE 8:**
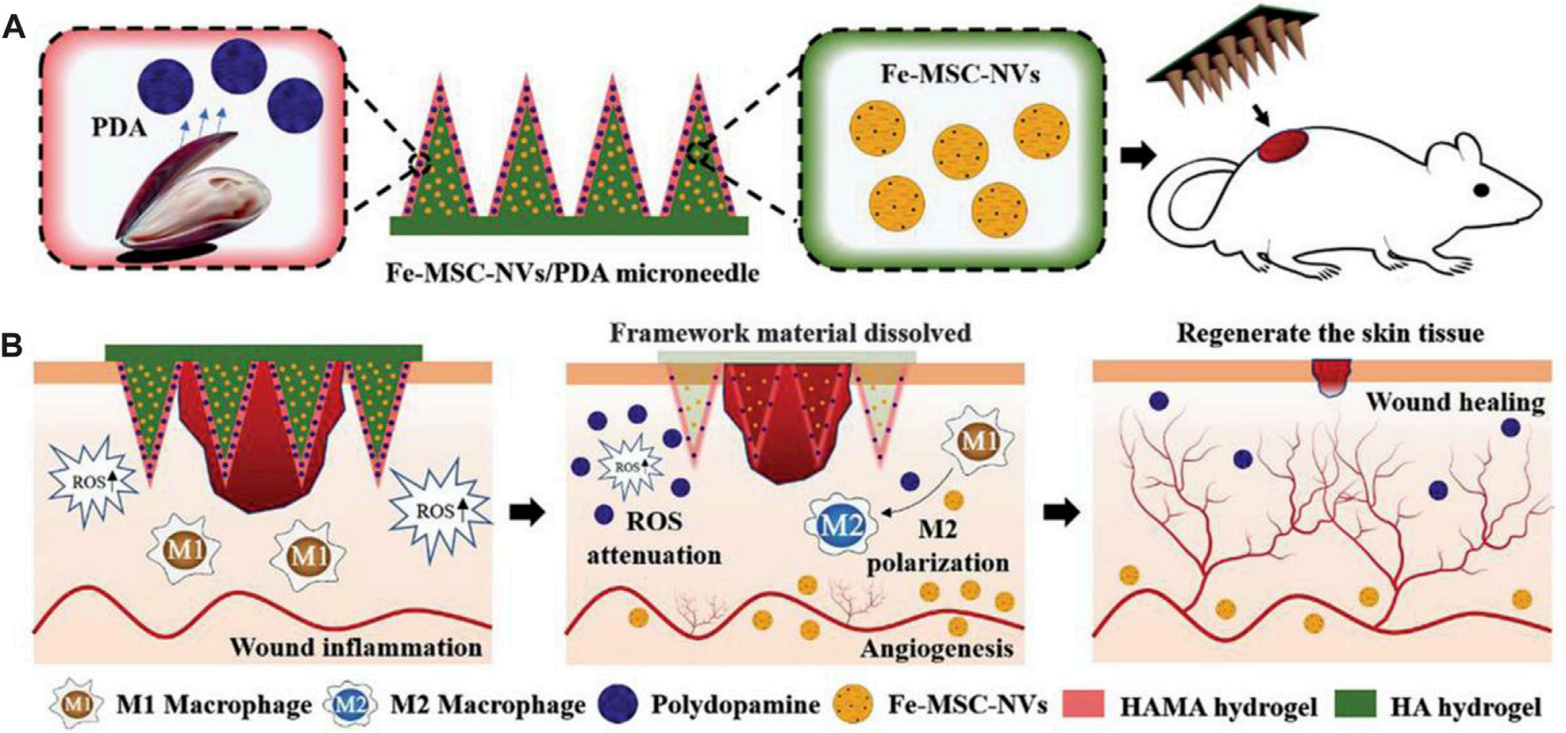
Schematic illustrations of the Fe-MSC-NVs/PDA MN patch for diabetic wound healing: **(A)** schematic of the Fe-MSC-NVs/PDA MN patch; **(B)** schematic of the wound closure process (Ma et al., 2022).

Ma et al. (2022) proposed a novel core-shell hyaluronic acid (HA) MN patch that encapsulates iron-mesenchymal stem cell-derived artificial nanovesicles (Fe-MSC-NVs) and polydopamine nanoparticles (PDA NPs) for wound healing (Figure 8). The Fe-MSC-NVs, containing multifunctional therapeutic cytokines, were encapsulated within the HA core at the tip of the MN to accelerate angiogenesis. PDA NPs were encapsulated within the shell of methacrylic acid-modified hyaluronic acid (HAMA) to overcome the detrimental effects of ROS-derived oxidative stress. The results demonstrated that Fe-MSC-NVs significantly enhanced the migration, proliferation, and tube formation of human umbilical vein endothelial cells (HUVECs). Furthermore, the combination of PDA NPs and Fe-MSC-NVs further promoted M2 macrophage polarization, thereby suppressing wound inflammation. In *in vivo* experiments, the Fe-MSC-NVs/PDA MN patch showed excellent efficacy in diabetic wound healing.

These findings emphasize the potential of engineered extracellular nanovesicles as a promising approach for skin wound repair in regenerative medicine. Therefore, a mixed system containing growth factors, MSCs, EVs, and other antioxidant chemical substances can effectively treat non-healing wounds through various pathways. We have summarized the above studies in Table 3.

**TABLE 3:** Application of stem cells and cell products in accelerated wound healing.

Treatment	Models	Result	Reference
MSCs-EGF-Cur B	Rat	Significant promotion of wound healing, collagen protein synthesis, formation of granulation tissue, and growth of new blood vessels	Mohanty and Pradhan (2020)
PVA-TPA-GM-CSF	Mice	Decreased ROS levels and upregulated M2-phenotype macrophages around the wound to promote wound closure	(Zhao et al., 2020)
BMSCs	Mice	Improved wound closure, increased reepithelialization and angiogenesis	de Mayo et al. (2017)
CKD-ATMSCs	Mice	CKD-AT-MSCs are functionally deficient and the wound healing process is delayed	Khanh et al. (2017)
ADSCs-exo	Rat	Oxidative stress in diabetic wounds is significantly reduced, promoting the proliferation of epithelial cells and hair follicles	Shiekh et al. (2020b)
Fe-MSC-NVs	Rat	Promote the polarization of M2 macrophages, inhibit inflammation and accelerate wound healing	Ma et al. (2022)

Reactive oxygen species play an important role in many biological functions. Excessive ROS and oxidative stress can induce a strong inflammatory response, interfere with tissue repair, disrupt tissue repair, and disrupt the immune system. Implantation of wound dressings after skin wound can also increase oxidative stress as well as increase inflammatory cell types. To date, numerous ROS scavengers have been proposed, including systemically delivered nanoparticles, therapeutic drugs, and biomaterials for inflammation regulation and tissue repair. Currently, biomaterials with the inherent ability to regulate ROS by removing ROS have been designed, including free radical scavenging polymers, therapeutic drugs, antioxidants, enzymes, and nanomaterials, and have been used for skin tissue regeneration and cell therapy. This chapter details the practical application of these ROS-scavenging biomaterials in skin wound. These materials can release biosignaling molecules, growth factors and therapeutic drugs through damage microenvironment and cell-mediated ways, which help regulate ROS, reduce oxidative stress, and regulate tissue repair. These biological materials that regulate ROS require further attention from the scientific community.

## 6 Future and prospects

In the field of skin repair, the application of ROS removal materials shows great potential and provides many interesting directions for future research and clinical applications. First, the development of new ROS removal materials will be the focus of attention. In recent years, advances in nanotechnology, materials science, and bioengineering have opened up new opportunities for the design and preparation of highly efficient ROS-removing materials. For example, by manipulating the surface structure and chemical functional groups of materials, higher ROS removal efficiency and selectivity can be achieved. In addition, the development of functionalized biomaterials that can not only clear ROS but also provide functions such as growth factors and cell adhesion matrix to facilitate the skin repair process will also become a promising research direction. Second, combination treatment strategies will play an important role in skin repair. Combining ROS removal materials with other therapeutic strategies allows for a more comprehensive and synergistic therapeutic effect. For example, combined with technologies such as phototherapy, gene therapy or drug delivery, more precise treatments can be achieved, providing customized treatment options for different types of skin injuries and diseases. This combined treatment strategy is expected to improve treatment outcomes and accelerate the skin repair process. Third, translating ROS scavenging materials from *in vitro* studies to *in vivo* experiments and clinical applications is an important goal for the future. Although some progress has been made, there are still some challenges to successfully applying ROS removal materials to clinical treatment. For example, issues such as material stability, biocompatibility and drug delivery need to be addressed. Future research should focus on addressing these questions and validating the safety and efficacy of ROS-clearing materials through clinical trials and clinical trials.

In addition, an in-depth understanding of the mechanism of ROS in different skin pathological processes is also an important research direction. By studying the mechanisms of ROS production and signaling and their influence on cell function in different diseases, ROS clearance materials can be better designed and optimized to achieve more precise treatments. Personalized treatment strategies are also a prospective research direction in the future by targeting the characteristics of specific diseases and individuals to design and apply ROS clearance materials to achieve better therapeutic outcomes.

In summary, ROS removal materials have broad prospects and potential in the field of skin repair. Future research will focus on the development of novel materials, the application of combination therapy strategies, the challenges of clinical translation, mechanistic research, and personalized therapy. With continued innovation and progress, ROS removal materials are expected to become an important tool for skin repair, providing patients with better treatment options and efficacy.
